# Thalamocortical interactions reflecting the intensity of flicker light–induced visual hallucinatory phenomena

**DOI:** 10.1162/netn_a_00417

**Published:** 2025-03-03

**Authors:** Ioanna A. Amaya, Till Nierhaus, Timo T. Schmidt

**Affiliations:** Neurocomputation and Neuroimaging Unit, Department of Education and Psychology, Freie Universität Berlin, Berlin, Germany; Charité – Universitätsmedizin Berlin, Einstein Center for Neurosciences Berlin, Berlin, Germany; Humboldt-Universität zu Berlin, Berlin School of Mind and Brain, Berlin, Germany

**Keywords:** Visual hallucinations, Flicker light stimulation, Altered states of consciousness, Thalamocortical connectivity, Thalamic nuclei, Functional connectivity

## Abstract

Aberrant thalamocortical connectivity occurs together with visual hallucinations in various pathologies and drug-induced states, highlighting the need to better understand how thalamocortical interactions may contribute to hallucinatory phenomena. Flicker light stimulation (FLS) at 10-Hz reliably and selectively induces transient visual hallucinations in healthy participants. Arrhythmic flicker elicits fewer hallucinatory effects while delivering equal amounts of visual stimulation, together facilitating a well-controlled experimental setup to investigate the neural correlates of visual hallucinations driven by flicker rhythmicity. Using rhythmic and arrhythmic FLS during fMRI scanning, we found that rhythmic FLS elicited stronger activation in higher order visual cortices compared with arrhythmic control. Consistently, we found that rhythmic flicker selectively increased connectivity between ventroanterior thalamic nuclei and higher order visual cortices, which was also positively associated with the subjective intensity of visual hallucinatory effects. As these thalamic and cortical areas do not receive primary visual inputs, it suggests that the thalamocortical connectivity changes relate to a higher order function of the thalamus, such as in the coordination of cortical activity. In sum, we present novel evidence for the role of specific thalamocortical interactions with ventroanterior nuclei within visual hallucinatory experiences. Importantly, this can inform future clinical research into the mechanistic underpinnings of pathologic hallucinations.

## Author Summary

Flicker light stimulation induces an intense transient experience of elementary visual hallucinations, such as colored geometric patterns, that has phenomenal similarities to the visual experience induced by psychedelic drugs or during psychopathology. During fMRI scanning, we found that increased connectivity between ventroanterior thalamic nuclei and higher order visual cortices was associated with the reported intensity of the flicker-induced visual effects. Our results suggest that the role of thalamocortical hyperconnectivity during hallucinatory experiences may relate to a higher order function of the thalamus, such as the regulation of cortical activity. This novel finding results from a highly controlled experimental setup, which can be extended to inform the mechanistic underpinnings of hallucinatory experiences during psychopathology.

## INTRODUCTION

The thalamus is a subcortical structure that has diverse functions in filtering sensory information, regulating cortical excitability, and integrating information across cortical networks (Halassa & Sherman, 2019; Hwang, Bertolero, Liu, & D’Esposito, 2017; Sherman & Guillery, 2006; Shine, Lewis, Garrett, & Hwang, 2023). The various thalamic functions are served by different thalamic nuclei, whereby first-order nuclei (e.g., lateral geniculate nuclei [LGN]) mainly relay and filter sensory information and higher order nuclei (e.g., ventroanterior, reticular nuclei) are primarily involved in the coordination of activity and information availability across cortices. Thalamocortical hyperconnectivity has been observed in a range of pathologies and altered states of consciousness (Fort et al., 2024), such as psychosis (Anticevic, Cole, et al., 2014; Anticevic, Yang, et al., 2014), Charles Bonnet syndrome (Ffytche, 2008), and during psychedelic experiences (F. Müller, Dolder, Schmidt, Liechti, & Borgwardt, 2018; Preller et al., 2019; Rieser et al., 2024). In some cases, thalamocortical hyperconnectivity has been correlated to the intensity of hallucinatory experiences (Anticevic, Yang, et al., 2014; F. Müller et al., 2018). However, there is not yet a consensus on which thalamic nuclei display changes in connectivity. A better understanding of which thalamic nuclei become hyperconnected can help unravel the functional relevance of thalamocortical hyperconnectivity during visual hallucinatory experiences.

To investigate which thalamic nuclei display thalamocortical hyperconnectivity, it is crucial to identify an experimental tool that can selectively elicit hallucinatory experiences without additional confounding effects. For example, while thalamocortical connectivity during psychotic states and psychedelic experiences has been extensively investigated (Anticevic, Cole, et al., 2014; Anticevic, Yang, et al., 2014; Avram et al., 2020, 2022; Bedford et al., 2023; Ferri et al., 2018; F. Müller et al., 2017; Pizzi et al., 2023; Preller et al., 2019), hallucinatory experiences within these populations co-occur with other physiological effects, such as long-term compensatory changes for psychotic patients (Lieslehto et al., 2021) and widespread systemic effects arising from pharmacological intervention (Hirschfeld, Prugger, Majić, & Schmidt, 2023; Luppi et al., 2021; Schmid et al., 2015). This means that the described neural correlates are not necessarily specific to the visual experience. On the contrary, flicker light stimulation (FLS) can reliably and selectively induce visual hallucinatory effects in healthy participants via closed-eye application of stroboscopic light. The flicker-induced effects are mainly constituted of elementary visual hallucinations, referring to the perception of geometric patterns and colors devoid of semantic content (Amaya, Behrens, Schwartzman, Hewitt, & Schmidt, 2023; Bartossek, Kemmerer, & Schmidt, 2021). These elementary phenomena are also frequently reported during psychedelic experiences (Prugger, Derdiyok, Dinkelacker, Costines, & Schmidt, 2022; Schmidt & Berkemeyer, 2018), for those with Charles Bonnet syndrome (Jan & del Castillo, 2012), and for some patients with psychosis (van Ommen, van Laar, Cornelissen, & Bruggeman, 2019). Therefore, utilizing FLS allows the assessment of specific neural effects occurring during visual hallucinations that are also experienced in other pathologies and pharmacologically induced states.

Flicker rhythmicity and frequency interact to produce different intensities of hallucinatory effects (Amaya, Behrens, et al., 2023), with rhythmic 10-Hz FLS eliciting the strongest visual effects. Recent research has identified increased connectivity between anterior, ventral, and mediodorsal thalamic nuclei and visual cortices during 10-Hz FLS compared with 3-Hz FLS (Amaya, Schmidt, et al., 2023). However, comparing FLS-induced neural effects across flicker frequencies introduces differences in the amount of visual stimulation. Therefore, we recently developed a frequency-matched arrhythmic flicker sequence that eradicates the confound of stimulation intensity and significantly reduces visual effects (Amaya, Behrens, et al., 2023). This allows us to test for the rhythmicity-dependent effects of FLS that drive differences in the subjective experience.

In this study, we implemented rhythmic 10-Hz FLS and frequency-matched arrhythmic FLS to test which thalamic nuclei display changes in functional connectivity during FLS-induced visual hallucinations. Additionally, we used a block design with rhythmic and arrhythmic flicker sequences to assess differential changes in whole-brain cortical activation, where we expected that rhythmic FLS would elicit stronger activation clusters in higher order visual cortices compared with arrhythmic stimulation. To assess the resting-state functional connectivity, we utilized a fine anatomic parcellation of the thalamus (Rolls et al., 2020) and visual functional regions (Wang et al., 2015). We hypothesized there to be a rhythmicity-dependent effect of 10-Hz FLS on connectivity changes between higher order visual cortices and thalamic nuclei, primarily anterior, ventral, and mediodorsal nuclei, as found previously (Amaya, Schmidt, et al., 2023). The stronger activation and connectivity patterns during rhythmic 10-Hz FLS compared with arrhythmic would therefore be associated with differences in visual hallucination intensity.

## METHODS

### Participants

Twenty participants took part in the experiment (15 men, five women; *M*_age_ ± *SD* = 29 ± 7.9 years). Participants were recruited through word of mouth and student mailing lists within Freie Universität Berlin. Eighteen participants were right-handed and two were left-handed according to the Edinburgh Handedness Inventory (Oldfield, 1971; mean laterality quotient for right-handers [*M* ± *SD*] = 64.7 ± 18.7; laterality quotient for left-handers = −60, −20). Inclusion criteria involved no acute mental disorders, no current consumption of psychotropic medication, and previous experience with FLS to minimize the risk of aversive effects. Participants gave their written consent before proceeding with the experiment. The experimental design and materials were approved by the ethics committee at Freie Universität Berlin (application reference: 085/2021).

### Experimental Materials and Setup

**Figure 1.**
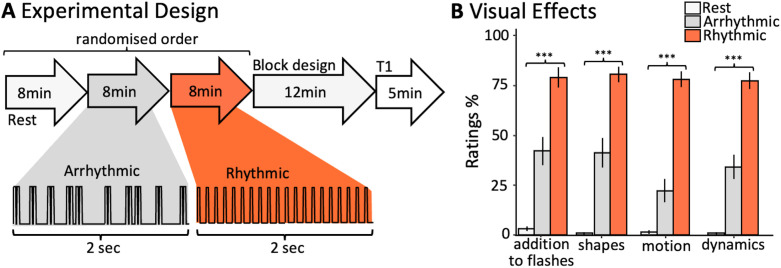
(A) The scanning session comprised of three 8-min resting-state scans, namely, (a) closed-eye rest in darkness, (b) closed-eye rest with arrhythmic FLS, and (c) closed-eye rest with 10-Hz rhythmic FLS, presented in a fully randomized order. After each resting-state scan, the participants responded to four questions about their subjective experiences via the scanner intercom system. Thereafter, the participants underwent a 12-min run with eyes closed, where rhythmic and arrhythmic FLS sequences were presented in a block design (see the Methods section for details). Each block lasted 20 s, followed by a 20-s baseline interval. An anatomical scan was performed before participants were released from the scanner. (B) Mean scores of questionnaire items for each experimental condition, indicating that flicker rhythmicity significantly affects the reported intensity of phenomena additional to flashes, perceived shapes, motion, and experience dynamics. Stars represent the significance of a one-way repeated-measures ANOVA to test the effect of experimental condition on subjective ratings (*p* < 0.001 marked with ***). Error bars represent the standard error of means.

A stroboscope lamp with twelve 4,500 k J2 6 V light-emitting diodes was used to generate the light stimulation, capable of delivering a maximum of 10,360 lumens (Lumenate Growth Ltd., Bristol, United Kingdom). For study implementation, the lamp was programmed to deliver 8,125 lumens, which is approximately 80% of its total capacity. The lamp was placed outside the scanner, perpendicular to the direction of the scanner bore (in place of the usual projector) and reflected by the MRI mirror system into the scanner bore. Convex lenses were placed above the participants’ eyes to refocus the light and increase comparability of the setup to previous studies (Amaya, Behrens, et al., 2023; Montgomery, Amaya, & Schmidt, 2024). The lamp was interfaced with an Arduino (v1.8.16) to deliver FLS at two rhythmicity levels, namely, rhythmic 10-Hz, which consisted of periodic light stimulation following a 0.3 duty cycle (30 ms on, 70 ms off), and arrhythmic flicker (Figure 1A). The arrhythmic flicker sequence used pairs of high-frequency flashes embedded within longer intervals sampled from an exponential probability distribution (*M* = 70 ms; see the Arrhythmic_pairs_ condition from Amaya, Behrens, et al., 2023, for more details on the arrhythmic sequence). Rhythmic 10-Hz FLS was applied to induce the highest intensity of visual hallucinatory phenomena, while arrhythmic stimulation elicits significantly fewer hallucinatory effects (Amaya, Behrens, et al., 2023).

### Study Design and Procedure

MRI scanning was conducted at the Center for Cognitive Neuroscience Berlin, Freie Universität Berlin. The scanning session comprised of three 8-min resting-state scans, namely, (a) closed-eye rest in darkness, (b) closed-eye rest with 10-Hz rhythmic FLS, and (c) closed-eye rest with 10-Hz arrhythmic FLS, presented in a fully randomized order (Figure 1A). After each resting-state scan, the participants rated four questions (see below) about their subjective experiences via the scanner intercom system. Thereafter, the participants underwent a 12-min functional scan with eyes closed, during which a block design with six conditions was applied: (a) darkness, (b) constant light, (c) 3-Hz rhythmic FLS, (d) 3-Hz arrhythmic FLS, (e) 10-Hz rhythmic FLS, and (f) 10-Hz arrhythmic FLS. Each block lasted 20 s, followed by a 20-s baseline interval. Each condition was presented three times within three blocks of all six conditions, where, within each block, the order of conditions was randomized. Subsequently, a 5-min anatomical scan was performed before the participants were released from the scanner and experiment.

### FLS-Induced Phenomenology

Phenomenological aspects of FLS-induced effects were retrospectively assessed using four questionnaire items from an adapted version of the Stroboscopic Visual Experience Survey, which were previously identified as most characteristic of the subjective experience (Amaya, Behrens, et al., 2023). The items were as follows: “Did your visual experience consist of anything in addition to blackness and/or flashes?”; “Did your visual experience consist of geometric formations including points, lines, shapes and/or patterns?”; “Did your visual experience include motion? Such as patterns or shapes that moved across, around or within your visual field?”; and “Did your visual experience continuously change or evolve over time?” Participants were asked to give an integer rating between 0 (*no, not at all*) and 100 (*yes, very much*).

### fMRI Scanning

Participants were scanned using a 3T MAGNETOM Prisma Fit MRI scanner equipped with a 64-channel head coil (Siemens Healthineers, Erlangen, Germany). BOLD sensitive images were acquired using a T2*-weighted echo planar imaging sequence (48 axial slices acquired interleaved with multi-slice factor 3; in-plane resolution = 2.5 × 2.5 mm; slice thickness = 2.5 mm; flip angle = 70°; TR = 1,500 ms; TE = 33 ms). To acquire structural images, a T1-weighted magnetisation-prepared rapid gradient-echo sequence was used (TR = 1,930 ms; TE = 3.52 ms; flip angle = 8°; voxel size = 0.8 × 0.8 × 0.8 mm). Cushioned head supports were fitted to minimize head motion.

### MRI Data Preprocessing

Data were preprocessed using SPM12 (www.fil.ion.ucl.ac.uk/spm/). Slice time correction and realignment was applied to the functional data before spatial normalization to MNI152 space using unified segmentation in SPM12, which included reslicing to an isometric 2-mm voxel size (Ashburner & Friston, 2005).

### Cortical Activation Analysis

The realigned and normalized data were smoothed with an 8-mm full width at half maximum (FWHM) Gaussian kernel. Statistical analysis was performed according to a standard general linear model approach. The first-level design was specified to model the six task conditions (rhythmic 10-Hz, arrhythmic 10-Hz rhythmic 3-Hz, arrhythmic 3-Hz, constant light, and no light), and independent regressors and motion parameters were modeled in six regressors of no interest. On the first level, we computed contrasts against implicit baseline for the rhythmic 10-Hz, arrhythmic 10-Hz rhythmic 3-Hz, arrhythmic 3-Hz, constant light, and no light conditions. These contrasts were entered into a second-level flexible factorial design, as implemented in SPM12, which included modeling of the subject factor (Gläscher & Gitelman, 2008). The second-level contrasts 10-Hz rhythmic > implicit baseline and 10-Hz rhythmic > 10-Hz arrhythmic were computed. All activation differences are reported at *p* < 0.05, family-wise error (FWE)-corrected on the cluster level, unless stated differently. Thresholded statistical parametric maps were rendered on a standard 3D brain template using MRIcroGL v1.2.20220720b (https://www.nitrc.org/projects/mricrogl/). Unthresholded T-maps can be found at https://neurovault.org/collections/FEKKFQRX/. All coordinates are reported in the MNI space of SPM12.

### Resting-State fMRI Analysis

A low-pass Butterworth filter of 0.15-Hz was applied to realignment parameters to filter out factitious motion induced by magnetic field perturbations during respiration (Fair et al., 2020; Gratton et al., 2020; Power et al., 2018), before calculating the framewise displacement (FD) using BRAMILA tools (Power, Barnes, Snyder, Schlaggar, & Petersen, 2012). Volumes that exceeded a threshold of 0.4 mm were masked during following analysis steps (“scrubbing”). The mean FD across scans and participants was 0.08 ± 0.04 (*M* ± *SD*), and the mean percentage of volumes scrubbed for each scan was 0.44 ± 0.67 (*M* ± *SD*), with the maximum percentage being 3.13%. Principal component analysis (CompCor) was performed using the DPABI toolbox (toolbox for Data Processing & Analysis for Brain Imaging; https://rfmri.org/dpabi) within the cerebrospinal fluid (CSF)/white matter mask to estimate nuisance signals (Behzadi, Restom, Liau, & Liu, 2007). Anatomical masks for CSF and white and gray matter were derived from tissue probability maps provided in SPM12. Smoothing was performed with a 3-mm FWHM Gaussian kernel to retain high spatial specificity for small regions of interest (ROIs; Chen & Calhoun, 2018; Mikl et al., 2008; Pajula & Tohka, 2014). The first five principal components of the CompCor analysis, six head motion parameters, linear and quadratic trends, and the global signal were used as nuisance signals to regress out associated variance. All analyses were additionally run without global signal regression and included in the Supporting Information. Finally, the toolbox REST (www.restfmri.net) was used for temporal bandpass filtering (0.01–0.08-Hz).

Anatomical ROIs were extracted using the automated anatomical labeling (AAL3) atlas (Rolls et al., 2020) for the thalamus and a volume-based maximum probability map (MPM) of visual topography (Wang et al., 2015) for cortical areas. Using probability maps of V1 and V2, overlapping ROIs at the midline were resolved by assigning voxels to the region with the highest probability. Of the thalamic ROIs, the nucleus reuniens is only 8 mm^3^ and was excluded from our analyses. Additionally, when the MPM was resliced to 2 mm^3^, hMST and IPS5 were no longer present in one hemisphere. For consistency, these ROIs were removed bilaterally.

For each ROI, mean BOLD time courses were extracted, and temporal ROI-to-ROI Pearson correlations were calculated and, subsequently, Fisher *z* transformed. We took the mean of correlation coefficients for ipsilateral and contralateral connections to give one bilateral functional correlation coefficient for each pair of ROIs. For all ROI-to-ROI pairs, we took the group-level average of correlation coefficients for closed-eye rest, rhythmic, and arrhythmic resting-state scans and then computed the differences via matrix subtraction. A mask was applied so that only significant differences (paired *t* test, *p* < 0.05, false discovery rate (FDR) corrected with the Benjamini–Hochberg method [Benjamini & Hochberg, 1995]) were shown.

Baseline connectivity and unthresholded connectivity change matrices can be found in Supporting Information Figures S1–S6. To exclude the potential effects of white matter tracks close to the thalamus, we conducted two control analyses. First, we reran our main analysis without any spatial smoothing, which revealed highly similar results (see Supporting Information Figure S7). Second, we tested for potential effects of the optical tracts (Wang et al., 2023); that is, we tested whether the signal from the optic chiasm was correlated to the signals of thalamic nuclei. When contrasting rhythmic against rest and rhythmic against arrhythmic conditions, paired *t* tests (*α* = 0.05) revealed no significant changes in connectivity between the optic chiasm ROI and any thalamic nuclei. Taken together, these control analyses support the spatial specificity of the presented analyses and do not indicate that there is relevant signal leakage from optic white matter tracts.

### Linear Mixed Modeling

To test the relationship between subjective experience and connectivity changes, we ran linear mixed-effects models, with subjective ratings as a fixed effect and ROI-to-ROI functional connectivity as the dependent variable. Participants were included as a random effect, and the models were fit using the restricted maximum likelihood method. As the ratings of each questionnaire item were highly correlated with each other (*ρ* = 0.84–0.95), ratings were taken as the average of the four questionnaire items for each participant for each condition. Akaike information criterion values were similar for models with random intercepts only and models with random intercepts and slopes; therefore, models were run with random intercepts and slopes to account better for intersubject variability. Additionally, we ran linear mixed models using subjective ratings as the fixed effect and the percent signal change of peak voxels as the dependent variable. Percent signal changes were extracted from peak voxels in the clusters identified by the 10-Hz rhythmic > 10-Hz arrhythmic contrast using the rfxplot toolbox implemented within SPM12 (Gläscher, 2009). To test the underlying assumptions of linear mixed modeling, normal Q–Q plots were used to assess whether residuals of the linear mixed model fit followed a Gaussian distribution. Homoscedasticity was confirmed by plotting the residuals against predicted values and observing no pattern in the noise distribution. The assumption of independence was addressed by including participants as a random effect in the model.

## RESULTS

### Subjective Ratings

Repeated-measures analyses of variance (ANOVAs) identify a significant effect of condition on ratings of “Did your visual experience consist of anything in addition to blackness and/or flashes?” (*F*(2, 38) = 88.94, *p* < 0.001); “Did your visual experience consist of geometric formations including points, lines, shapes and/or patterns?” (*F*(1.5, 28.0) = 91.43, *p* < 0.001); “Did your visual experience include motion? Such as patterns or shapes that moved across, around or within your visual field?” (*F*(2, 38) = 122.93, *p* < 0.001); and “Did your visual experience continuously change or evolve over time?” (*F*(2, 38) = 85.11, *p* < 0.001), where all conditions produced ratings that were significantly different from each other for each item, with 10-Hz rhythmic FLS generating the highest ratings, followed by arrhythmic and closed-eye rest (Figure 1B). As ratings were not normally distributed (e.g., tendency for a positive skew during closed-eye rest conditions), we additionally confirmed these results using repeated-measures Friedman nonparametric tests to test the effects of experimental condition on perceived motion (*χ*^2^(2) = 38, *p* < 0.001), dynamics (*χ*^2^(2) = 34.2, *p* < 0.001), perceived patterns and shapes (*χ*^2^(2) = 36.1, *p* < 0.001), and seeing something additional to flashes (*χ*^2^(2) = 35.6, *p* < 0.001).

### Flicker-Induced Cortical Activation

**Figure 2.**
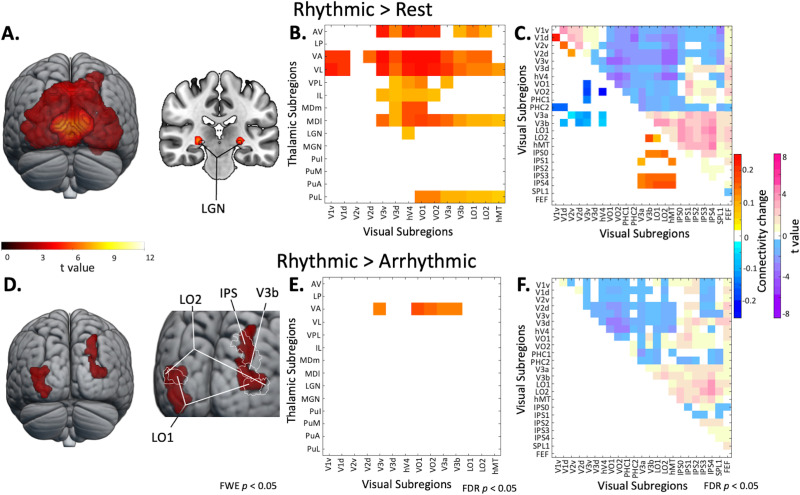
(A) Cortical activation clusters for the rhythmic 10-Hz against implicit baseline contrast (FWE *p* < 0.05 on the cluster level) reveal increased activation across the occipital cortex. When *p* < 0.05 is FWE-corrected on the peak level, the rhythmic 10-Hz against implicit baseline contrast additionally reveals bilateral LGN activation. (B) Thalamocortical functional connectivity matrix showing the contrast of rhythmic 10-Hz FLS against closed-eye rest reveals an increase in connectivity of anterior, ventral, mediodorsal, and lateral pulvinar nuclei with visual cortices, which replicates previous findings (Amaya et al., 2023). Unthresholded connectivity matrices are presented in the Supporting Information. (C) Corticocortical functional connectivity matrix showing the contrast of rhythmic 10-Hz FLS against closed-eye rest reveals two clusters of hyperconnectivity within early visual cortices as well as between IPS and higher order visual areas (e.g., LO1, V3a). There is an additional cluster of hypoconnectivity between early visual cortices and higher order visual areas. (D) The rhythmic 10-Hz against arrhythmic contrast reveals that the activation dependent on flicker rhythmicity is specific to higher order visual cortices. (E) Thalamocortical functional connectivity matrix showing the contrast of rhythmic 10-Hz FLS against arrhythmic FLS. Connectivity between ventroanterior thalamic nuclei and higher order visual areas (e.g., VO1) is significantly higher during rhythmic FLS. (F) Corticocortical functional connectivity matrix of the contrast rhythmic 10-Hz FLS against arrhythmic FLS showing no significant differences in connectivity. All connectivity matrices are thresholded at *p* < 0.05 with FDR correction.

To reveal the cortical activation induced by the FLS condition, we computed the second-level contrasts 10-Hz rhythmic > implicit baseline and 10-Hz rhythmic > arrhythmic FLS. The rhythmic > implicit baseline cluster revealed increased activity in the entire occipital cortex, as shown in Figure 2A, with *p* < 0.05 FWE-corrected on the cluster level. The activation cluster (*K*_E_ = 17,507) extends bilaterally across the occipital cortex (peak: *x* = 0, *y* = −82, *z* = 4, *t* value = 11.87; subpeaks *x* = 10, *y* = −76, *z* = 10, *t* value = 11.05 and *x* = 10, *y* = −88, *z* = 4, *t* value = 10.46) with peaks in medial ventral occipital areas. Additionally, one cluster was revealed in the intraparietal area (peak: *x* = 24, *y* = −48, *z* = 46, *t* value = 6.19, *K*_E_ = 387). On the peak level, there were small clusters in bilateral LGN regions of the AAL3 atlas (right peak: *x* = 26, *y* = −24, *z* = −2, *t* value = 7.09, *K*_E_ = 45; left peak: *x* = −26, *y* = −24, *z* = −2, *t* value = 7.77, *K*_E_ = 62). Meanwhile, the rhythmic > arrhythmic contrast unveiled a specific increase in the activation of bilateral higher order visual cortices (left cluster *K*_E_ = 691; right cluster *K*_E_ = 564), with *p* < 0.05 FWE-corrected on the cluster level (left peak: *x* = −34, *y* = −80, *z* = 10, *t* value = 4.21; left subpeaks: *x* = −32, *y* = −78, *z* = 4, *t* value = 4.14 and *x* = −28, *y* = −86, *z* = −10, *t* value = 4.06; right peak: *x* = 32, *y* = −76, *z* = 12, *t* value = 4.29; right subpeaks: *x* = 24, *y* = −70, *z* = 46, *t* value = 3.84 and *x* = 28, *y* = −72, *z* = 34, *t* value = 3.78). The left peak is in LO2, and the right peak is not within a region of the MPM but has a 12% probability of being part of LO2. One right subpeak (*x* = 24, *y* = −70, *z* = 46) is part of the IPS0 region of the MPM (Figure 2D). Unthresholded T-maps, accessible via https://neurovault.org/collections/FEKKFQRX/, show relatively symmetrical bilateral activation. Additionally, 10-Hz arrhythmic > implicit baseline, 10-Hz rhythmic > 3-Hz rhythmic, and 3-Hz rhythmic > 3-Hz arrhythmic contrasts were computed and included in Supporting Information Figure S8.

### Flicker-Induced Functional Connectivity

We plotted connectivity matrices with 14 thalamic ROIs from the AAL3 atlas (Rolls et al., 2020) and 14 cortical ROIs from an MPM of visual topography (Wang et al., 2015). The rhythmic > rest contrast shows an increase in connectivity between visual cortices and anterior, ventral, and mediodorsal nuclei (Figure 2B), which replicates previous findings (Amaya, Schmidt, et al., 2023). Please note that connectivity between LGN and visual cortices was also increased during rhythmic FLS; however, this did not survive FDR correction due to existing baseline connectivity. Unthresholded connectivity matrices, which also include additional ROIs (e.g., intraparietal sulci), can be found in Supporting Information Figures S4–S7. Figure 2C displays corticocortical functional connectivity matrices of rhythmic FLS against closed-eye rest in darkness (*p* < 0.05, FDR-corrected), where there are two clusters of hyperconnectivity within early visual cortices and between intraparietal sulci and higher order visual areas (e.g., LO1, V3a) and an additional cluster of hypoconnectivity between early visual cortices and higher order visual areas. This replicates findings from a previous dataset (Amaya, Schmidt, et al., 2023). When testing the rhythmic against the arrhythmic condition, we found a specific increase in connectivity (*p* < 0.05, FDR-corrected) between ventroanterior and higher order visual cortices (e.g., VO1) during rhythmic FLS (Figure 2E). We found no significant differences in corticocortical connectivity changes between rhythmic and arrhythmic FLS (Figure 2F).

### Linear Mixed Modeling

We ran linear mixed models with subjective ratings of hallucination intensity as a fixed effect to determine if the reported intensity of visual effects (i.e., mean of four questionnaire items; see the Methods section) could predict changes in functional connectivity between thalamocortical ROI-to-ROI pairs. We applied a Bonferroni-corrected significance threshold (0.05/198). We found that subjective ratings significantly predicted connectivity between ventroanterior nuclei and VO1 (*p* = 0.0001, *β* = 0.004; CI [0.0017, 0.0053]; *R*^2^_adj_ = 0.51) and V3a (*p* = 0.0002, *β* = 0.002; CI [0.0011, 0.0036]; *R*^2^_adj_ = 0.28). Additionally, subjective ratings significantly predicted connectivity between ventrolateral nuclei and VO1 (*p* = 3.9e−0.5, *β* = 0.004; CI [0.0021, 0.0055]; *R*^2^_adj_ = 0.68), V3a (*p* = 1.5e−0.5, *β* = 0.003; CI [0.0017, 0.0041]; *R*^2^_adj_ = 0.0.36), and V3b (*p* = 0.0001, *β* = 0.002; CI [0.0012, 0.0033]; *R*^2^_adj_ = 0.39). We also tested the relationship between subjective ratings and the percent signal change in the peak voxels revealed by the 10-Hz rhythmic > 10-Hz arrhythmic activation contrast. Here, we only found a minor association between subjective ratings and activation differences in the peak voxel within LO2 of the left hemisphere (*p* = 0.027) and the right-hemispheric peak voxel (*p* = 0.035), of which neither survives correction for multiple comparisons. Together, this shows that the subjective intensity of hallucinatory effects is more associated with ventral thalamic connectivity to higher order visual cortices than the amount of cortical activation.

## DISCUSSION

In this study, we tested for thalamocortical connectivity changes related to the subjective intensity of visual hallucinations induced by FLS; that is, we tested for effects on rhythmicity, which influences the intensity of subjective experiences. First, we tested the effects of 10-Hz flicker rhythmicity on cortical activation, where we found a stronger activation of higher order visual cortices during 10-Hz rhythmic FLS compared with arrhythmic control. Next, we identified increased connectivity between higher order thalamic regions and visual cortices during 10-Hz rhythmic FLS. With this, we replicated the results of a previous study (Amaya, Schmidt, et al., 2023), where data were acquired from a different MRI scanner and using a different FLS lamp. Extending from the previous study, we found a specific rhythmicity-dependent connectivity change between ventroanterior thalamic nuclei and higher order visual cortices, where rhythmic FLS induced a stronger increase in functional connectivity. Consistently, rhythmic FLS also led to higher ratings of hallucination intensity across all measured domains, replicating findings from a previous study using the same questionnaire items (Amaya, Behrens, et al., 2023). Linear mixed modeling revealed a positive relationship between thalamocortical connectivity and the reported intensity of hallucinatory effects, while the relationship between subjective ratings and cortical activation did not survive correction for multiple comparisons. In sum, we show that increased connectivity between ventroanterior thalamic nuclei and higher order visual cortices relates to the increased intensity of visual hallucinatory effects during 10-Hz rhythmic flicker stimulation.

### Rhythmicity-Specific Activation in Visual Cortices

When assessing differences in cortical activation, we found that rhythmic 10-Hz FLS led to stronger activation specifically in higher order areas of the visual cortex compared with arrhythmic FLS. This supports results from a previous fMRI study (Ffytche, 2008), which administered 8-Hz FLS alongside two control conditions: FLS with matched frequency but low brightness and FLS where the flashes were presented in pairs. The activation during 8-Hz FLS against low-brightness control covered the occipital cortex similarly to our rhythmic 10-Hz FLS against baseline contrast. As the stimulation frequencies (i.e., 8-Hz, 10-Hz) lie within the alpha frequency range, which is known to elicit the most hallucinatory effects (Allefeld, Pütz, Kastner, & Wackermann, 2011; Amaya, Behrens, et al., 2023), it is expected that the activation results between studies are comparable. The activation clusters revealed in contrasts against both control conditions were in higher order visual areas, such as the V5 complex; occipitotemporal cortices; and intraparietal sulci (Ffytche, 2008). However, it must be noted that these findings were derived from a fixed-effects model with six subjects. In our study, we refine these results by using a larger sample and a statistical model that accounts for the subject factor. Moreover, we developed and utilized an improved control condition consisting of arrhythmic flashes (Amaya, Behrens, et al., 2023) accompanied by subjective ratings. Therefore, we provide more robust evidence for the increased activation of higher order visual areas during hallucination-inducing FLS.

The visual areas that showed stronger activation during rhythmic 10-Hz FLS compared with arrhythmic FLS included LO1 and LO2, which are selective for orientation and shape perception, respectively (Grill-Spector et al., 1998; Malach et al., 1995; Silson et al., 2013); V3b, which is motion sensitive (Barton & Brewer, 2017); and the right intraparietal sulcus, which is part of the dorsal attention network (Vossel, Geng, & Fink, 2014) but has also been found to respond selectively to simple geometric forms (Sereno & Maunsell, 1998). Together, the basic visual features that activate these cortical regions make up the key features of elementary visual hallucinations, namely, colored geometric patterns that move around the visual field. Indeed, the 10-Hz rhythmic FLS was shown to increase ratings of perceived motion, shapes, and patterns, found here and previously using the same questionnaire items (Amaya, Behrens, et al., 2023), suggesting that the activation of these visual cortical areas may contribute to the hallucinatory perception of elementary features.

Computational modeling has suggested that elementary visual hallucinations arise from perturbations of cortical excitation and inhibition in the primary visual cortex (B. G. Ermentrout & Billock, 2018; G. B. Ermentrout & Cowan, 1979; Rule, Stoffregen, & Ermentrout, 2011), which can occur when rhythmic visual stimulation interacts with ongoing intrinsic oscillations. This was recently supported by an empirical study finding that standing waves are generated in the mouse visual cortex in response to rhythmic FLS (Gulbinaite et al., 2024). However, while this framework focuses on activity within primary visual cortices, most theories of conscious phenomenal experience require interactions with higher order cortices, such as local recurrent interactions (Northoff & Lamme, 2020; Seth & Bayne, 2022), feedback interactions across the visual hierarchy (Corlett et al., 2019), or long-range interactions with supramodal regions (e.g., global neuronal workspace theory [Dehaene & Changeux, 2011; Mashour et al., 2020]). In line with this, our data capture an increased BOLD signal in higher order visual cortices during rhythmic FLS, which is in line with their functional contribution to color, motion, and shape perception (as described above), and confirm previous reports (Ffytche, 2008). In this regard, while the interplay of cortical excitation and inhibition within primary visual cortices may initiate subsequent neural and phenomenal effects, our data emphasize the involvement of higher order visual cortices as a relevant correlate of the subjective experience.

### Thalamocortical Connectivity With Ventroanterior Thalamic Nuclei

We found that rhythmic FLS led to selective increases in connectivity between ventroanterior nuclei and higher order visual cortices, which was also associated with the intensity of reported visual effects. This presents a novel finding that refines previous work, which had found that 10-Hz FLS increased thalamocortical connectivity with anterior, ventral, and mediodorsal nuclei compared with 3-Hz FLS (Amaya, Schmidt, et al., 2023), by removing confounds of stimulation intensity resulting from differences in flicker frequency. It should be noted that the arrhythmic condition also induced a weak increase in connectivity between ventroanterior thalamic nuclei and visual cortices (see Supporting Information Figure S3), which aligns with the fact that hallucinatory effects are also weakly induced during arrhythmic FLS, shown here (Figure 1B) and previously (Amaya, Behrens, et al., 2023). This is highlighted by the strong relationship between ratings of hallucination intensity and connectivity between ventroanterior (and ventrolateral) thalamic nuclei and higher order visual cortices.

Ventroanterior nuclei are higher order thalamic nuclei (Hwang et al., 2017), suggesting that thalamocortical hyperconnectivity within hallucinatory experiences relates to a higher order function of the thalamus, such as coordinating cortical activity. Ventroanterior thalamic nuclei contain a high density of matrix cells that diffusely project to cortical layer 1 (Jones, 2001; E. J. Müller et al., 2020; Whyte, Redinbaugh, Shine, & Saalmann, 2024). The activation of cortical layer 1 reduces perceptual thresholds (Takahashi et al., 2020), which has been further speculated to drive subjective experiences in the absence of sensory stimuli, such as during dreaming (Aru, Siclari, Phillips, & Storm, 2020). This is encompassed by a recent theory emphasizing the role of cortical interactions with higher order thalamic nuclei to form conscious experiences (Aru, Suzuki, Rutiku, Larkum, & Bachmann, 2019). However, it must be noted that, to date, there is little evidence showing an anatomic connection between ventral thalamic nuclei and visual cortices. This suggests that the connectivity between ventroanterior and visual cortices may also be mediated by the thalamic reticular nucleus, which receives cortical and subcortical inputs and exerts inhibitory control over thalamocortical neurons (Halassa & Sherman, 2019; Li et al., 2020; Pinault, 2004). Furthermore, ventral nuclei (including ventroanterior and ventrolateral divisions) were found to display above-average connectivity to the right lateral visual network (Kumar, Beckmann, Scheffler, & Grodd, 2022), which is involved in shape and motion perception (Smith et al., 2009), demonstrating the feasibility that increased interactions between ventral thalamic nuclei and higher order visual cortices are involved in FLS-induced visual effects.

Hallucinatory experiences can be elicited through various methods; however, the underlying neural state may be similar regardless of the induction technique. For this reason, comparing findings from research using different hallucination induction methods can shed light on the shared neural effects and reinforce their likely relevance within visual hallucinatory experiences. For example, one study found that LSD induced hyperconnectivity between ventral and pulvinar thalamic nuclei and the sensorimotor network (Pizzi et al., 2023), while another found that LSD induced hyperconnectivity between ventral thalamic nuclei and auditory-sensory cortical networks (Avram et al., 2022). While psychedelic experiences involve additional phenomenal and physiological effects, the shared connectivity pattern with FLS effects consistently increased cortical connectivity with ventral thalamic nuclei. This further supports that connectivity with ventral thalamic nuclei may be most relevant in the formation of visual hallucinatory experiences.

The FLS parameters (e.g., frequency, rhythmicity) that optimally induce hallucinatory effects can provide valuable insights into the possible underlying mechanisms of visual hallucinations. Research has consistently shown that stimulation around 10-Hz elicits the strongest hallucinatory effects, which coincides with the EEG alpha frequency range (Allefeld et al., 2011; Amaya, Behrens, et al., 2023; Amaya, Schmidt, et al., 2023; Schwartzman et al., 2019; Smythies, 1959, 1960). This may relate to the role of alpha oscillations in gating information flow by regulating neuronal firing in the gamma frequency (Haegens et al., 2011; Jensen et al., 2012; Spaak et al., 2012), that is, cross-frequency coupling (Canolty & Knight, 2010; Klimesch et al., 2007; Kosciessa et al., 2021; Wang et al., 2012). This alpha oscillation is said to be under the control of rhythmically bursting thalamocortical neurons (Womelsdorf et al., 2014). Using open-eye stimulation of rhythmic flicker (e.g., via methodology described in Dowsett et al., 2020a), it was found that alpha visual flicker can modulate perceptual awareness (van Dijk et al., 2008; Gulbinaite et al., 2017a, 2017b), facilitate multisensory integration (Dowsett et al., 2020a, 2020b), and drive neural entrainment (Jaeger et al., 2023; Notbohm & Herrmann, 2016; Notbohm, Kurths, & Herrmann, 2016). It remains likely that similar underlying mechanisms may drive the visual effects of closed-eye FLS, whereby the homogeneity of sensory input gives way for hallucinations to emerge. Therefore, based on knowledge gained from open-eye flicker paradigms, it can be suggested that alpha frequency FLS elicits hallucinatory effects by interacting with the endogenous alpha oscillations that modulate neuronal excitability in the visual cortex.

### Limitations and Future Directions

It is known that FLS can also induce other phenomenal effects, such as complex visual imagery (e.g., hallucinatory perception of realistic scenes [Bartossek et al., 2021; Schwartzman et al., 2019]), enhancing the emotional response to music (Montgomery et al., 2024), and altering the sense of self (Schwartzman et al., 2019). The underlying mechanism of these additional phenomenal effects is pivotal to the potential clinical applications of FLS. For example, alterations in the sense of self may be linked to hypoconnectivity across the default mode network, as found with psychedelic interventions (Gattuso et al., 2022; F. Müller et al., 2018). However, these complex phenomena tend to emerge during FLS sessions that were designed to be maximally immersive, which is usually achieved through varying flicker frequencies and brightness, as done in recreational settings. Future studies could utilize FLS sequences that are optimized to induce immersive experiences in order to assess the neural correlates of these additional phenomenal qualities. This would improve the comparability of FLS to other states, such as psychedelic experiences, while also lending to the clinical potential of FLS.

Furthermore, in this study, questionnaire items were designed to give a retrospective evaluation of the intensity of visual experiences. Here, each item was answered with a percentage to represent the intensity of a phenomenal characteristic (e.g., patterns, motion) over an 8-min period. This means there is loss of information about the moment-to-moment characteristics of the visual experience. Future research should aim to develop a more fine-grained phenomenological assessment, perhaps allowing in-moment reports to capture the visual dynamics over time. This could help refine our understanding of how specific aspects of the subjective experience relate to neural changes.

It is well established that FLS in the alpha frequency range elicits the most intense visual hallucinatory effects (Allefeld et al., 2011; Amaya, Behrens, et al., 2023; Schwartzman et al., 2019; Smythies, 1960). Here, we administered 10-Hz FLS to be representative of the alpha frequency; however, recent research has found that using rhythmic flicker targeting the individual alpha frequency can lead to stronger effects of entrainment and changes in functional connectivity (Jaeger et al., 2023). Therefore, it is possible that accounting for the interindividual variation in responsivity to alpha stimulation could unravel a stronger subject-specific relationship between cortical activation, connectivity changes, and the reported visual effects.

In this study, we propose that the activation and connectivity differences induced by rhythmic and arrhythmic FLS are linked to differences in phenomenal experience. However, it remains possible that the neural effects are driven solely by flicker rhythmicity without being tied to phenomenal experiences. Still, as evidence indicates that FLS rhythmicity is critical for the intensity of the hallucinatory experience (Amaya, Behrens, et al., 2023; Ffytche, 2008), likely due to neural entrainment driving the effects (Jaeger et al., 2023; Notbohm & Herrmann, 2016; Notbohm et al., 2016; Schwartzman et al., 2019), there is reason to suggest that flicker rhythmicity and the visual effects are bound to each other. With this in consideration, while we do not present a causative link, our utilization of a highly controlled experimental induction of visual hallucinations offers, to our knowledge, the most direct link between specific thalamocortical interactions and subjective experiences of visual hallucinations currently available in the literature. Importantly, this can be used to inform further clinical research into the mechanisms of hallucinatory symptomology.

### Conclusion

In sum, we show that higher order visual cortices are specifically activated during rhythmic 10-Hz FLS compared with arrhythmic control. Furthermore, the rhythmicity of 10-Hz FLS selectively elicits an increase in functional connectivity between ventroanterior thalamic nuclei and higher order visual cortices. These connectivity and activation changes reflect the difference in subjective ratings of perceived motion, dynamics, and shapes during FLS. Our results suggest that the role of thalamocortical hyperconnectivity during hallucinatory experiences is linked to a higher order function of the thalamus, such as the coordination of cortical interactions.

## ACKNOWLEDGMENTS

We thank Lumenate Growth Ltd. (Bristol, United Kingdom) for generously providing experimental hardware free of charge.

## SUPPORTING INFORMATION

Supporting information for this article is available at https://doi.org/10.1162/netn_a_00417.

## AUTHOR CONTRIBUTIONS

Ioanna Alicia Amaya: Data curation; Formal analysis; Investigation; Methodology; Software; Visualization; Writing – original draft; Writing – review & editing. Till Nierhaus: Data curation; Methodology; Resources; Software; Writing – review & editing. Timo Torsten Schmidt: Conceptualization; Funding acquisition; Investigation; Methodology; Project administration; Resources; Software; Supervision; Writing – review & editing.

## FUNDING INFORMATION

The investigator-initiated study was financially supported by a donation from Lumenate Growth Ltd. to Freie Universität Berlin allocated to T.T.S. I.A.A. is a PhD candidate funded by the Einstein Center for Neurosciences, Charité Universitätsmedizin Berlin.

## DECLARATION OF COMPETING INTERESTS

Within the last 5 years, an unrestricted donation from Lumenate Growth Ltd. to Freie Universität Berlin was allocated to T.T.S. Lumenate Growth Ltd. played no role in study conceptualization, data collection, analysis, or decision to publish.

## Supplementary Material



## TECHNICAL TERMS

Parcellation: Division of the brain’s gray matter into nonoverlapping groups of voxels, often named regions of interest (ROIs).
Echo planar imaging: MRI acquisition technique that is sensitive to the blood oxygenation level dependent (BOLD) signal in the brain.
General linear model (GLM): A mass univariate statistical approach that determines how much each regressor (e.g., experimental condition) explains the signal variance of each voxel.
Resting-state fMRI: The measurement of spontaneous low-frequency fluctuations in the BOLD signal that correspond to the functional organization of brain networks.
Maximum probability map: A method for parcellating gray matter using the maximum probability that a given voxel belongs to a particular functional or anatomical region.
Linear mixed modeling: An extension of linear regression to include random effects that additionally account for sources of variance within sample subgroups.
ROI-to-ROI functional connectivity: The temporal dependence of BOLD time series between regions of interest, calculated via Pearson correlations.
